# Assessing the Radiological Risks Associated with High Natural Radioactivity of Microgranitic Rocks: A Case Study in a Northeastern Desert of Egypt

**DOI:** 10.3390/ijerph19010473

**Published:** 2022-01-01

**Authors:** Neveen S. Abed, Mohamed Abdel Monsif, Hesham M. H. Zakaly, Hamdy A. Awad, Mahmoud M. Hessien, Chee Kong Yap

**Affiliations:** 1 Geochemical Exploration Department, Nuclear Materials Authority, El-Maadi, Cairo P.O. Box 530, Egypt; nova848@yahoo.com (N.S.A.); drmonsifzaky@hotmail.com (M.A.M.); 2 Institute of Physics and Technology, Ural Federal University, 620002 Yekaterinburg, Russia; 3 Physics Department, Faculty of Science, Al-Azhar University, Assiut Branch, Assiut 71524, Egypt; 4 Institute of Earth Sciences, Southern Federal University, Zorge St., 40, 344090 Rostov-on-Don, Russia; hamdiawaad@gmail.com; 5 Geology Department, Faculty of Science, Al-Azhar University, Assuit Branch, Assuit 71524, Egypt; 6 Department of Chemistry, College of Science, Taif University, P.O. Box 11099, Taif 21974, Saudi Arabia; hessienmahmoud@yahoo.com; 7 Department of Biology, Faculty of Science, Universiti Putra Malaysia (UPM), Serdang 43400, Malaysia

**Keywords:** Wadi Al-Baroud, high activity concentration, radiological impacts, microgranite, Egypt

## Abstract

This study aimed to evaluate the radiological hazards of uranium (^238^U), thorium (^232^Th), and potassium (^40^K) in microgranitic rocks from the southeastern part of Wadi Baroud, a northeastern desert of Egypt. The activity concentrations of the measured radionuclides were determined by using a gamma-ray spectrometer (NaI-Tl-activated detector). The mean (^238^U), (^232^Th), and (^40^K) concentrations in the studied rocks were found to be 3680.3, 3635.2, and 822.76 Bq/kg, respectively. The contents in these rocks were elevated, reaching up to 6.3 wt%. This indicated the alkaline nature of these rocks. The high ratios of Th/U in the mineralized rocks could be related to late magmatic mineralization, suggesting the ascent of late magmatic fluids through weak planes such as faults and the contact of these rocks with older granites. The present data were higher than those of the United Nations Scientific Committee on the Effects of Atomic Radiation (UNSCEAR) guideline limits. All the radiological hazard results indicated high human health risks. This confirmed that this area is not radiologically safe, and care must be taken when working in this area. This study showed that the area under investigation had high U content suitable for uranium extraction that could be used in the nuclear fuel cycle.

## 1. Introduction

**Figure 1:**
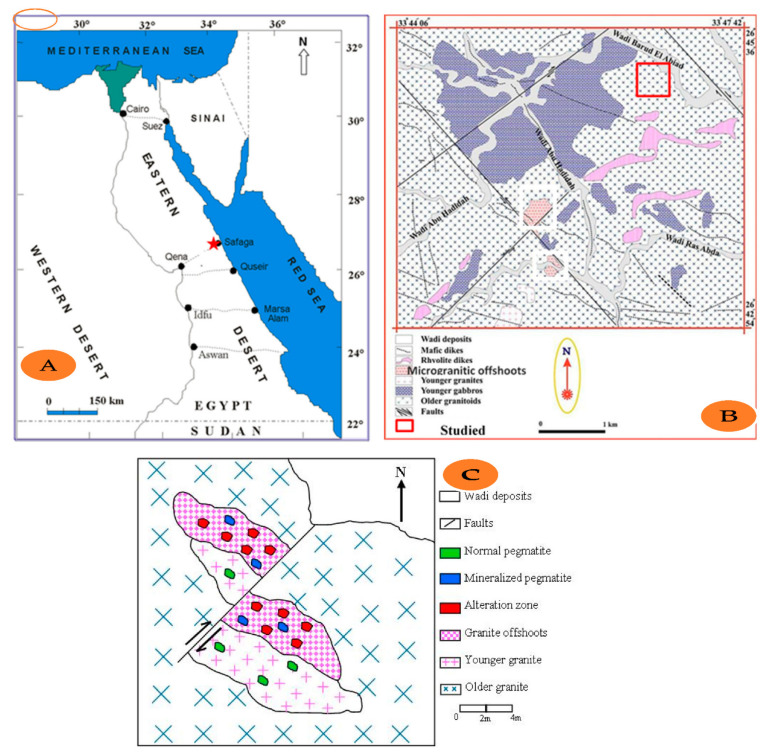
(**A**) Sampling map of the studied area, (**B**) Geological map of the Abu Hadeida area (Modified after Omran, 2015) and (**C**) Map of the studied area.

Uranium (^238^U), thorium (^232^Th), and potassium (^40^K) are some of the most incompatible elements, and they are concentrated in granitic rocks in the continental earth crust. U and Th occur mainly in accessory minerals such as monazite, orthite or allanite, zircon, sphene, and ammonium [1,2]. Most radioactivity in a basement rock of Egypt is found in granites and associated pegmatites. The presence of accessory minerals such as zircon, thorite, uranotorite, monazite, and allanite contributes to the high activity of these rocks [3]. The estimated mean values of radium equivalent in red and black jasper veins in the El-Misikat region are 1823.641 Bq/kg higher than the world’s recommended maximum value [4]. The mineralization of Nb-40 Ta in Egypt’s eastern desert has a direct connection to granite [5]. Omran, 2014, Studied, in detail, the highly radioactive microgranite dike-like bodies present in the studied area. He concluded that microgranites are alkali feldspar granite composed mainly of potash feldspars, mainly perthites and microcline, quartz, little sodic plagioclase, and biotite. Secondary uranium minerals (uranophane and curite), thorium minerals (thorite), and U- and Th-bearing minerals (Zircon, allanite, columbite, samarskite, xenotime, monazite, kasolite, and titanite) are identified. Other nonradioactive minerals such as magnetite, goethite, hematite, cronstedtite, pyrite, fluorite, and garnet are also identified [6]. Omran et al., 2015: the normal granite and the mineralized granite (microgranitic offshoots) are alkali feldspar granite, and essentially consist of potash feldspar (perthite, microcline perthite, and microcline), quartz, and rarely, mica. Zircon, allanite, kasolite, and uranophane as accessory minerals with thorite minerals [7]. The mineralized microgranite’s HFSE and total REE (TREE) content are unusual in less altered and normal granites. For example, the TREEs increase from 28.5 to 3373.8 ppm, with more enrichment with heavy rare earth minerals relative to light rare earth minerals. In mineralized microgranite, the HFSEs increase relative to those in the less altered microgranite, especially thorium, which increases 546-fold in the former compared to its original content in the latter. The magma chamber’s final magma ring at Abu Hadeida involved the formation of microgranitic veins, a process associated with highly developed fluids rich in Na and F. These fluids caused thermo-hydrothermal changes diffused along separate fluid pathways in the dams and fine veins containing an abundance of allanite, Zircon, apatite, and rare earth element content. The compound REEs were refilled with F in liquids, then re-deposited in dams and microscopic veins along weak levels, resulting in the formation of the mineralized zone [8]. Abd El Monsif, 2020, states that Abu Hadeida granite emerged from a magma rich in the concentrations of elements and minerals of high economic value; this granite, especially, was exposed to many tectonic movements that led to more of these important minerals, which make Abu Hadeida granite one of the most promising granites in Egypt. It also confirms that this granite is one of the most promising types in Egypt, containing high concentrations of uranium and thorium, which leads us to confirm that this granite emanated from a magma rich in minerals, including radioactive minerals that may be sourced from the magma molten or by the following processes, including hydrothermal solutions that worked on the precipitation of those minerals in a form easy to extract [9]. This work utilized a NaI(Tl) scintillation detector to evaluate the concentrations of radionuclides in the studied localities. Abd El Monsif, M., 2021, states that there are two types of pegmatite found in the Abu Hadeida area, the first is the mineralized pegmatite that is related to the microgranitic offshoots with several types of mineralization and the second one is the normal pegmatite related to the normal granite with no evidence of mineralization [10]. Apogranite is a type of granite that is thought to be a one-of-a-kind metasomatic granitoid [11]. One of the most important factors influencing the spatial variations of ^226^Ra, ^40^K, and ^232^Th in granite rocks is variations in trace element distribution [12]. The study area is restricted to the Northern Eastern Desert 20 km west of Safaga City between the Red Sea and the Nile Valley (Figure 1A). The area is easily accessible via the newly created part of Qena Safaga by an asphalt road that runs directly north of the area to Wadi Baroud. The area is bordered by the latitudes 26°42′54″ and 26°45′36″ N and the longitudes 33°44′06″ and 33°47′42″ E (Figure 1B). The radioactivity in the studied area is related to the microgranite offshoots present in minor faults. In the southern part of the Abu Hadeida district, several extensive radiometric and mineralogical studies have been carried out [6,13,14]. Anomalous radioactive mineralizations were first discovered in the Abu Hadeida area by Omran (2005) [13]. These anomalous radioactivities in the Abu Hadeida area are mainly connected to several mineralized microgranite offshoots, which intruded into the older granitoid rocks [6] (Figure 1A). This work aimed to evaluate the radiological hazards due to natural radioactivity in the microgranitic rocks from the southeastern part of Wadi Baroud in the northeastern desert of Egypt. The detailed sketched map of the studied area (Figure 1C) shows different rock units represented by younger granite, older granite, and granite offshoots.

On the other hand, several alteration zones are found in the granite offshoots with clear mineralization; also two types of pegmatite are found in the studied area, the first is the mineralized pegmatite related to the granite offshoots, and the second is the normal pegmatite related to the normal younger granite with no evidence of mineralization [10].

## 2. Methodology

### 2.1. Samples Preparation and Measurement

Nine microgranitic rocks samples were collected from the normal and altered microgranite at the southeastern part of Wadi Baroud, northeastern desert of Egypt in the summer of 2020. They were collected from the rocks and the alteration zones in the studied area in the summer of 2020. Egypt’s Nuclear Materials Authority (NMA) examined the cartographic material. The studied rocks represented a new occurrence for the mineralized microgranite dykes and veins that were previously recorded by Omran in 2014 and 2015 and Abdel Hamid et al. in 2018 [6,8,14].

Before the start of measurements, preparatory processes were carried out. Each sample was ground to approximately 60 mesh and mixed thoroughly to avoid non-uniform mineral distribution. The samples were then dried for one day at a temperature of 100 °C in an oven to remove all moisture. Thereafter, each sample was put in a standard size plastic container with the appropriate weight (300–350 g). These containers were carefully sealed to prevent contamination of the spectrometer. The well homogenization process will liberate the sample’s radon and improve the activity measurements. To compensate for radon losses, samples were held in sealed containers for at least 21 days before radon reverted to its original state and a radioactive equilibrium was achieved between ^238^U, ^232^Th, and their short-lived daughter products [15,16]. The relationship between the ^222^Rn accumulation percentage and the increase in time to reach a steady state was observed after about 38 days.

The gamma-spectrometric system, consisting of scintillation detector, a 7.6 cm × 7.6 cm NaI-(Tl) crystal, hermetically sealed by an aluminum photomultiplier tube, was housed in the Geochemical Research Department of the Egyptian Nuclear Materials Authority (NMA). An NaI detector has high efficiency and allows for a fast and precise detection of ^40^K, ^238^U, ^226^Ra, and ^232^Th concentrations in rock and soil samples. It is masked with its amplifier in a copper shield (0.6 cm thickness) against induced X-rays and a cylindrical lead chamber against the environmental radiations and connected to the “Accuspec card”. The Accuspec NaI plus 2k on board ADC, Amp and HVPS with 2k channel memory, which is attached to the PC computer board.

The concerned radionuclides U, Th, Ra, and K were measured using four energy regions of interest (ROI), which represented ^234^Th, ^212^Pb, ^214^Pb, and ^40^K, respectively. U and Th should be measured indirectly from their gamma-ray emitting daughters, ^234^Th and ^212^Pb, respectively. As a result, their material is expressed as eU (equivalent to uranium) and eTh (equivalent to thorium).

The energy stability of the channels of spectrometer differs with the change in the supply of power, voltage and with the change in the amplification characteristics of photomultiplier tube. Both of the parameters change according to the temperature. To know that the system accurately records the radiation energy of gamma for the radioactive elements, permanent calibration was determined by using radioactive calibration sources, ^137^Cs (661.6 KeV, setup in the channel 662) and ^57^Co (122.1 KeV, setup in the channel 122), as follows:

- Calibration was started with the ^137^Cs source (gain adjustment) and then with ^57^Co source (zero adjustment).
- The ^137^Cs source was repeated using a minimum procedure.

After the sample preparation processes, they were subjected to gamma analysis in accordance with the following steps:

(a) Equipment was setup using reference gamma-emitting sources (^137^Cs and ^57^Co) for energy calibration.
(b) Assaying of the samples using a long period net count, 1000 s for each, in the shielded environment and determination of the gross counts for U, Th, eU (Ra), and K at their selected energy regions as well.
(c) Determination of the background count rates in the selected energy regions (ROIs) for the laboratory with the detector.
(d) The registered spectrometric data (gross counts for eU, eTh, Ra, and K) for each sample were processed by using the computer program “ANALYSIS” with the use of background count rates, sample weight, time of measurement, and the preliminary sensitivity constants to determine the U, Th, and Ra concentrations in parts per million (ppm) and the K concentration in wt%.

The lower detection limit of U was 2 ppm, while the upper one was 2000 ppm for the low to medium grade samples. The detection limits for ore grade samples were greater than 2%. For uranium, the expected margin of error was between 10 and 15%. The detection limit for thorium was 0.6 ppm, with predicted errors ranging from 1 to 5%. The detection limit for radium was 0.4 ppm, with predicted errors ranging from 1 to 5%. Finally, the lower limit of detection of K was 0.1%, with predicted errors ranging from 1 to 5%.

### 2.2. Geochemical Analysis

At the Veritas Mineral Laboratories-ACME Lab in Canada, Vancouver, BC, Canada, the content of major and trace elements in less altered and mineralized rock samples was measured. To make about 20 g of pulp, the samples were ground to a 200 mesh. After multi-acid fermentation, the major oxides and several minor elements were analyzed using ICP-ES/MS. The difference in weight after ignition at 1000 °C was used to measure the ignition loss (LOI).

## 3. Data Treatment

### 3.1. Mobilization Type and Quantity

The type-inward migration or -outward migration and quantity of mobilized uranium in the studied rocks, as well as the rate of its mobilization, were calculated through several steps using the following equations of the Benzing Institute of China 1977 and China National Nuclear Corporation—CNNC, 1993:

(a) The paleo-uranium background:
  (1) $$U_{0}=\bar{eTh}\frac{\bar{U}}{Th}$$
  where *U*_0_ = 269.46, and $\bar{eTh}$ = the average Th content and $\frac{\bar{U}}{Th}$ = the average regional $\frac{U}{Th}$ ratio.
(b) The mobilized amount of uranium (*U_m_*): *U_m_* = *U_p_* − *U*_0_, where *U_m_* = 26.84 and *U_p_* is the average content of uranium.
  If *U_m_* > 0, the uranium migrated into the geological body during late evolution. If *U_m_* > 2, a significant amount of uranium migrated in.
(c) The mobilized uranium migration rate: P = $\frac{U_{m}}{U_{p}}$ × 100%, while P = 9.1%, As *U_m_* > 0, the uranium migrated in during the late evolution at a 9.1% migration rate. It was redistributed during or after alteration.

### 3.2. Radium Equivalent Activity 

According to Tufail et al., (1992), the radium equivalent ($Ra_{eq}$) activity can be expressed by the following formula:

(2) $$R_{eq}=A_{Ra}+\frac{10}{7}A_{Th}+\frac{10}{130}A_{K},$$

where $A_{Ra}$, $A_{Th}$ and $A_{K}$ are the specific activities of ^238^U(^226^Ra), ^232^Th, and ^40^K, in Bq kg^−1^, respectively [17,18].

### 3.3. Absorbed Dose Rate in the Air ($D_{r}$)

The $D_{r}$ was calculated by using the following equation:

(3) $$D_{r}\left({\mathrm{nGyh}}^{-1}\right)=0.462\times A_{U}+0.604\times A_{Th}+0.0417\times A_{K}$$

where $A_{U}$, $A_{Th}$, and $A_{k}$ are the average specific activities of ^226^Ra, ^232^Th, and ^40^K in Bq/kg, respectively [19,20,21]. The uniform distribution of the radionuclides ^238^U, ^232^Th, and ^40^K one meter above the ground’s surface was determined using the above equation.

### 3.4. Annual Effective Dose Equivalent (AEDE)

AEDE was estimated from the absorbed dose by utilizing the dose conversion factor of 0.7 Sv/Gy, an outdoor occupancy factor (OF) = 0.2, and an indoor occupancy factor (OF) = 0.8. The AEDE was calculated by using the following equation [19,20]:

(4) $$\mathrm{AEDE}\;(\mu \mathrm{Sv}/y)=D_{r}\;(\mathrm{nGy}/h)\;\times \;8760\;h\;\times \;\mathrm{OF}\;\times \;0.7\;\times \;10{-}^{3}\mathrm{Sv}/\mathrm{Gy}$$

where *D_r_* is dose rate in (nGy/h), 0.7 × 10^−3^ is the conversion coefficient in Sv/Gy, (0.2 × 24 h × 365.25 d) is the outdoor occupancy time, and (0.8 × 24 h × 365.25 d) is the indoor occupancy time [22]. These indices measure a risk of stochastic and deterministic exposures to exposed individuals. The recommended value of the AEDE is 0.48 mSv/y; the total AEDE_indoors_ and _outdoors_ should be less than one mSv/y [23].

### 3.5. External and Internal Hazard Index (H_ex_)

The *H_ex_* was calculated by using the following equation [24]:

(5) $$H_{ex}=\frac{A_{U}}{370}+\frac{A_{Th}}{259}+\frac{A_{K}}{4810}$$

Moreover, an internal exposure to ^222^Rn and its progeny was calculated by using the internal hazard index (H_in_) equation namely [25].

(6) $$H_{\mathrm{in}}=A_{U}/185+A_{Th}/259+A_{k}/4810$$

If both indices are less than unity, the radiation hazards are insignificant [26,27].

### 3.6. Gamma Activity Concentration Index (I_γ_)

The *I_γ_* was calculated by using the following equation [28]:

(7) $$I_{\gamma }=\frac{A_{U}}{150}+\frac{A_{Th}}{100}+\frac{A_{K}}{1500}$$

where *A_U_*, *A_Th_*, and *A_K_* are the activity concentrations of ^226^Ra (Bq/kg), ^232^Th (Bq/kg), and ^40^K (Bq/kg), respectively [29]. The values of e^238^U and e^232^Th, in ppm, as well as ^40^K, in %, were converted to activity concentration, in Bq kg^−1^, using the conversion factors given by the International Atomic Energy Agency, (IAEA, 1989) and by the Polish Central Laboratory for Radiological Protection (Malczewski et al., 2004).

The safety value for this index is ≤1 [30,31]. OECD experts suggested some criteria for defining different levels: representative, first enhanced, and second enhanced [28]. *I_γ_* equal to one is an upper limit, *I_γ_* ≤ 1 corresponds to 0.3 mSv/y, and *I_γ_* ≤ 3 corresponds to 1 mSv/y. Concerning various building materials, the ranges of *I_γ_* are as follows: Materials used in bulk-like bricks: *I_γ_* ≤ 0.5 − *I_γ_* ≤ 1S; Superficial materials and others: *I_γ_* ≤ 0.2 − *I_γ_* ≤ 6.

### 3.7. Annual Gonadal Dose Equivalent (AGDE)

The AGDE for the resident of a house was calculated by using the following equation [32]:

(8) $$\mathrm{AGDE}\left(\mu \frac{\mathrm{Sv}}{y}\right)=3.09\times {\;C}_{U}+418\times {\;C}_{\mathrm{Th}}+0.0317\times C_{K}$$

The world averages of AGDE in a house containing concentrations of ^238^U, ^232^Th, and ^40^K are 35, 35, and 370 mSv/y, respectively. The standard guideline for AGDE set by the United Nations Scientific Committee on the Effects of Atomic Radiation-UNSCEAR is 300 mSv/y.

### 3.8. Excess Lifetime Cancer Risk (ELCR)

The ELCR provides the probability of developing cancer over a lifetime at a given exposure level. ELCR was calculated by using the following equation [32]:

(9) $$\mathrm{ELCR}\left(m\frac{\mathrm{Sv}}{y}\right)=\;\mathrm{AEDE}\;\times \;\mathrm{DL}\times \mathrm{RF}$$

where DL is the duration of life (70 years average), and RF is the risk factor (Sv), i.e., the risk of dying from cancer per Sievert. For the public, the ICRP 106 used a value of RF = 0.05 for stochastic results.

### 3.9. Effective Dose Rate—D_organ_

The D_organ_ delivered to a particular organ was calculated by using the following equation [33]:

D_organ_ = AEDE × CF (10)

where CF is the organ to air dose conversion factor. The energies of interest in this work are 0.2–3 MeV, which is practically independent of energy. The main CF values for various organs and tissues are lung equal to 0.64, ovaries equal to 0.58, bone marrow equal to 0.69, testes equal to 0.82, and whole-body equal to 0.68.

## 4. Results

### 4.1. Radioactivity

**Figure 2:**
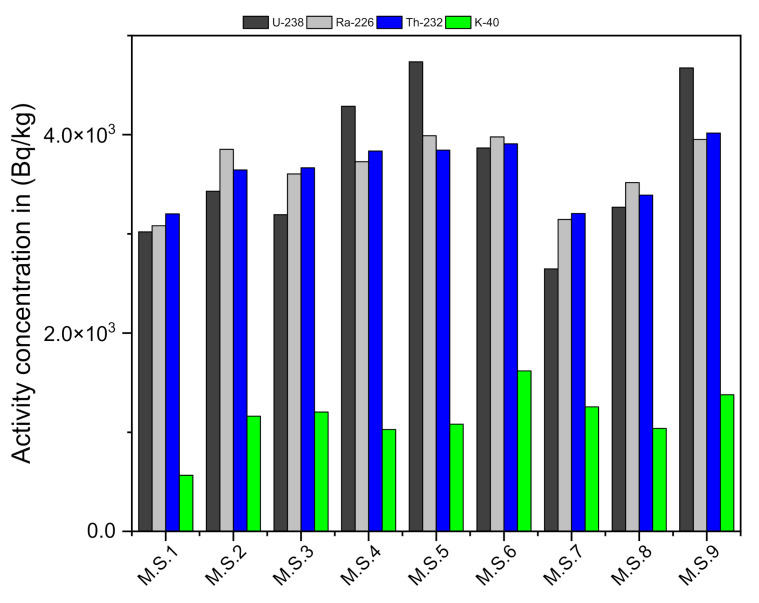
The activity concentrations (Bq/kg) of ^238^U, ^226^Ra, ^232^Th, and ^40^K in the nine samples of the Abu Hadeida microgranite.

The activity concentrations of ^238^U, ^226^Ra, ^232^Th, and ^40^K, in Bq/kg, and their ratios in Abu Hadeida microgranite, are presented in Table 1. The AC of the series of ^238^U, ^232^Th, and ^40^K in the nine samples of the studied anomalous microgranite are shown in Figure 2.

**Figure 3:**
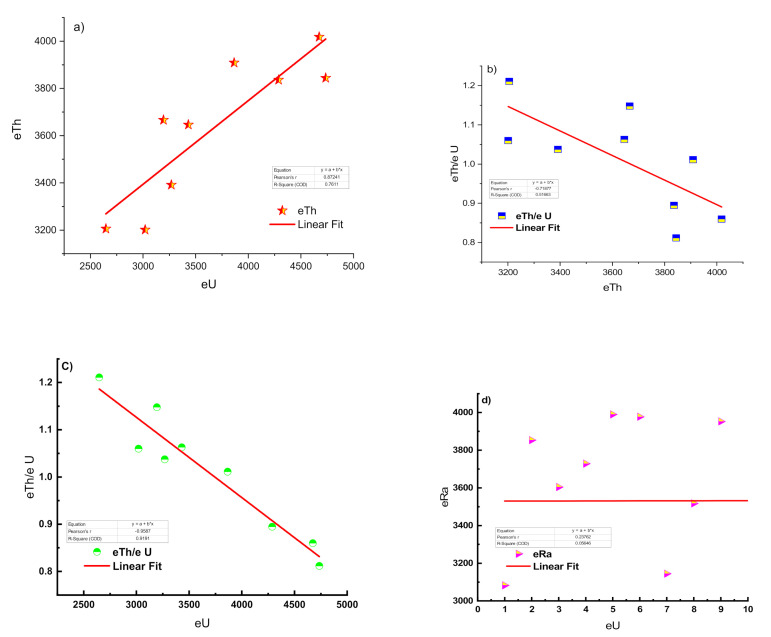

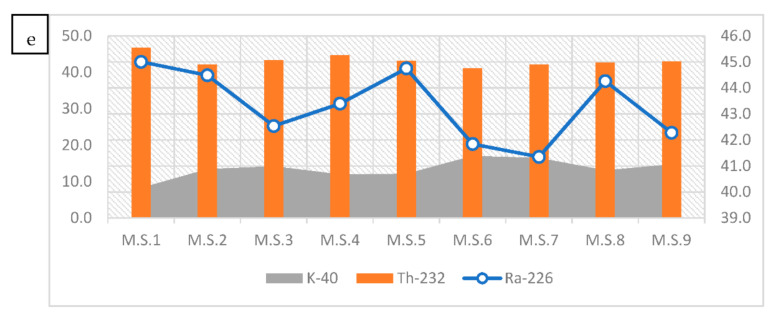
Bi-variation diagrams of (**a**) eU vs. eTh, (**b**) eTh/eU vs. eTh, (**c**) eU/eTh vs. eU, (**d**) eU vs. Ra eU, and (**e**) the relative contribution (%) of ^226^Ra, ^232^Th, and ^40^K to activity concentrations in every sample from the microgranite samples.

The ^238^U concentration ranges from 2647.4 to 4735.4 Bq/kg (mean: 3680.3 Bq/kg), while the ^232^Th concentration ranges from 3201.4 to 4017.9 Bq/kg (mean: 3635.2 Bq/kg). The ^226^Ra (eU) concentrations varied between 3082.4 and 3989.7 Bq/kg (mean: 3650 Bq/kg). The ^40^K concentration has a high concentration, reaching up to 6.3 wt% as well as these concentration values less than the values in the El-Missikat area that have a ^238^U contents average of 6304.6 Bq kg^−1^, and a ^4^K average of 42 Bq kg^−1^. (Awad H. A. et al., 2020 Applied radiation). The radioactive concentration in the study area was very highly related to radioactive bearing accessory minerals (Neeven et al., 2021). The geochemical behaviors of U and Th in the granitic rocks were examined by plotting the equivalent contents (eU and eTh), and Th/U ratios on variation, as shown in Figure 3. The variations of radioactive concentration especially depend on alteration zones and accessory minerals. In the present study, there are two reasons for high values of radioactive elements Firstly, hematitization and silicification features in addition to rare accessory and earth minerals in the study area (Neeven et al., 2021).

Figure 3a shows a positive correlation of U vs. Th, indicating the role of magmatic processes in both U and Th mineralization. The variation chart between eU and eTh content vs. eTh/eU ratio from Figure 3b,c, respectively, clarified that mineralization was not only magmatic but was also related to hydrothermal alteration processes. Typically, the concentrations of ^232^Th in natural rocks are three times greater than those of ^238^U. The effect of hydrothermal fluids on tonalite, granodiorite, monzogranite, syenogranite, and red and black siliceous veins was revealed by the scattering of data in cases of tonalite, granodiorite, monzogranite, syenogranite, and red and black siliceous veins [34]. In the Um-Tagir region, the relationship between U and Th/U showed a downward trend for granodiorite, monzogranite, and alkali feldspar granite, but an upward trend for tonalite. The data obtained for granite rocks showed that hydrothermal fluids influenced granodiorite, monzogranite, and alkaline feldspar granite, but no hydrothermal fluids influenced tonalite granite [35].

### 4.2. Radioactive Equilibrium

Two methods can determine the radioactive equilibrium in the studied area. The first method depends on the computation of the equilibrium factor (P), which is outlined as the ratio of the radiometrically determined U content (Ur) to the radium content: (P-factor = eU/R a(eU)). That was suggested by [36] and applied by El-Galy [37]. The second method uses data from the chemically analyzed U (Uc) and the radiometrically invented uranium (Ur). The ratio among the chemically and radiometrically invented U is known as the D-factor = Uc/Ur [38].

If both the P- and D-factors are greater than or less than one, uranium is being added or removed. Uranium removal and addition can be traced back to complex natural processes, such as a shift in the earth’s magnetic field that creates an imbalance. Groundwater may also have an effect on uranium reserves, allowing uranium to be leached from its original location and redeposited elsewhere. The loss of radon gas is another aspect that influences the equilibrium condition, owing to the solubility of radon in water and its leakage through pores, faults, and other cracks.

The measured U and Ra (eU) values were also plotted against each other to estimate the equilibrium state and provide an approximate estimate of the degree of agreement between these two values **(**Figure 3d). It was clear from Table 1 that the average value of the P-factor of the studied samples was more than one (P = 1), which pointed to equilibrium.

The use of the D-factor in determining the equilibrium state of the samples under study showed that all the samples under study contained more chemically analyzed ^238^U than radiometrically determined ^238^U, which reflected the non-equilibrium state characterized by the addition of ^238^U. The Uc/Ur ratio showed an average of 3.1, which meant an addition of ^238^U [38]. The relative contribution (%) of ^226^Ra, ^232^Th, and ^40^K to the activity concentration of every sample could be seen in Figure 3e. It was clear that the contribution activity of ^232^Th, ^226^Ra, and ^40^K in the samples differed from 41 to 46.7%, 41 to 45%, and 8.3 to 17%, respectively.

### 4.3. Uranium Mobilization and Migration

**Figure 4:**
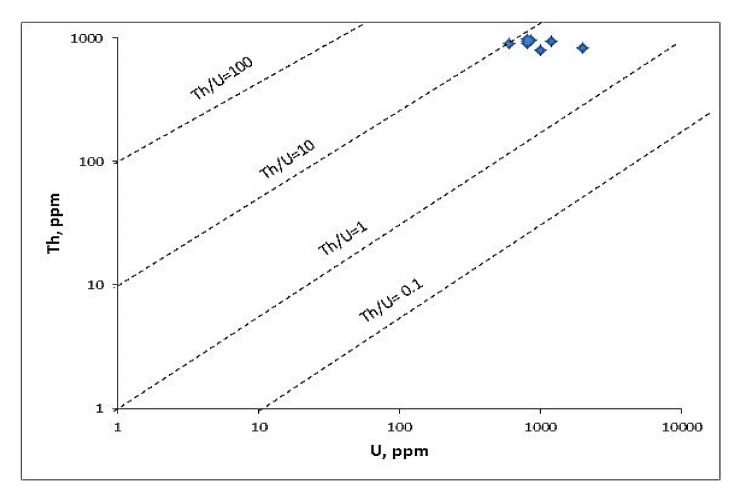
Th vs. U diagram for the studied Abu Hadieda microgranite.

A disturbance of this ratio indicates the depletion or enrichment of ^238^U. The eU and eTh variation diagrams with their ratios are used to estimate the amount of U mobilization within magmatic plutons [39,40]. Applying these diagrams to the studied rocks (Figure 3 and Figure 4) revealed that there were strong positive correlations between eU and eTh. At the same time, there was a negative correlation between them and their ratios (Figure 3a–d). This indicated the redistribution of the radioactive elements. The positive correlation between eU and eTh might be related to the accommodation of the two elements in the same minerals as thorite, uranothorite, Zircon, and xenotime. In tonalite and alkali feldspar granite, the U–Th binary diagrams showed a positive association between the two radioactive elements, but a negative correlation in granodiorite and monzogranite. Often, a strong association can be found with differentiation patterns, implying that radioactivity has a syngenetic basis. The negative correlation between ^232^Th and ^238^U, on the other hand, could indicate postmagmatic processes that were essential in the formation of ^238^U [34]. The positive relationship between ^232^Th and ^238^U may indicate a syngenetic origin for radioactivity in the ^238^U event; on the other hand, the negative relationship between ^232^Th and ^238^U may indicate that postmagmatic forms play a significant role in the ^238^U event [34,41].

**Figure 5:**
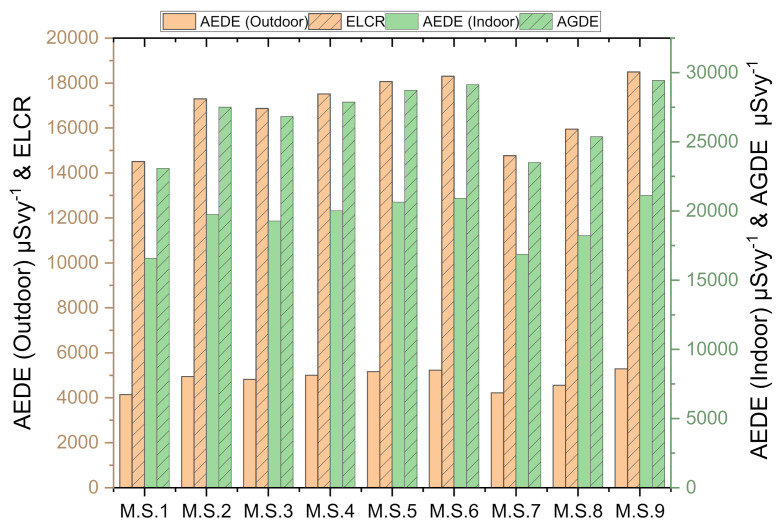
ELCR, AGDE, AEDE (indoor), and AEDE (outdoor) for all Abu Hadeida microgranite samples.

Overall statistics of ^238^U, ^226^Ra, ^232^Th, and ^40^K activity concentrations, radium equivalent, absorbed dose rate, gamma index, activity utilization index, excess-lifetime cancer risk (ELCR), annual gonadal equivalent (AGDE), AEDE_indoor_, AEDE_outdoor_, H_ex_, H_in_, and Clark ratio are presented in Table 2. The ELCR, AGDE, AEDE (indoor), and AEDE (outdoor) are displayed in Figure 5 for all the Abu Hadeida microgranite samples. The ELCR was found to have a mean value of 16,865 µSv/y (14,512–18,497 µSv/y). The AGDE values ranged from 23,084 to 29,441 µSv/y (mean: 26,834 µSv/y). Based on Table 2 and Figure 5, it was apparent that the AEDE_indoor_ was five times greater than the AEDE_outdoor_.

**Figure 6:**
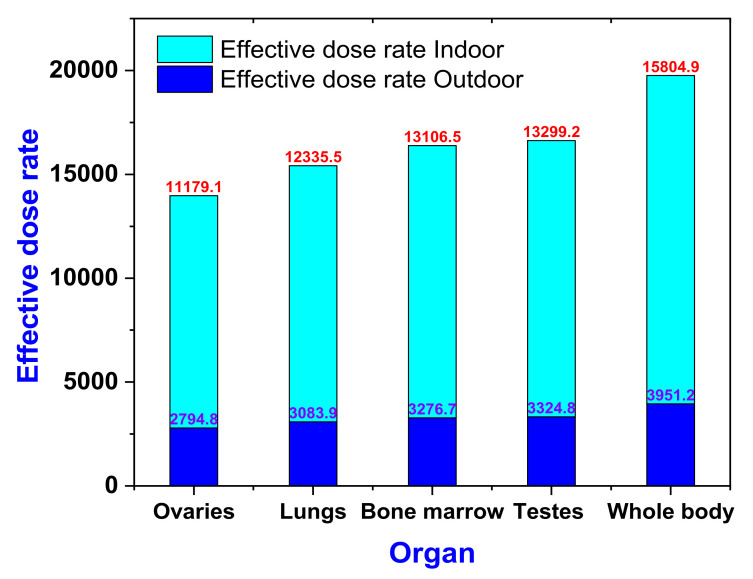
Effective dose rate (D_organ_) to different body organs and tissues in indoor and outdoor microgranite samples.

The values of AEDE_indoor_ and AEDE_outdoor_ were used to evaluate the effective dose rate (D_organ_) for different body organs and tissues, as shown in Figure 6. It was found that the highest effective dose rate was directed to the whole body, followed by testes, bone mallow, lung, and ovaries, in both indoor and outdoor.

## 5. Discussion

The Th/U ratios were <1 in the mineralized U samples (metasomatized). A few metasomatized samples had Th/U values higher than one [42]. The high ratios of Th/U (1.21) in mineralized rocks in the Abu Hadeida area could be related to the late magmatic mineralization of these granitic rocks, suggesting the ascent of late magmatic fluids through weak planes such as faults and the contact of these rocks with older granitoid. That was confirmed by field evidence. In the subduction zones, the content of Th of the calc-alkaline magma is habitually greater than the U content [43]. This could be explained in terms of the reduction and retention of both U and Th in the magma through the later stages of differentiation. If oxygen fugacity was low (lower than the Ni–NiO oxygen buffer), Th would be significantly enriched compared to U into the fluids differentiated magma [44]. Therefore, the reduction environment of late magmatic fluid(s) in the studied mineralized rocks explained the higher concentration of thorium. The overall ^232^Th concentration was higher than the global average as well as the average activities on Egyptian soil [34,41].

For comparison purposes, the ^238^U, ^232^Th, ^40^K, radium equivalent (Ra_eq_), absorbed dose rate (D_R_), and AEDE of the present study with those reported in the literature are presented in Table 3. In the Um Taghir area, [35] reported that the average activity (range) of the ^238^U (^226^Ra) series, ^232^Th series, and ^40^K were 15.7 (8.7–25.7), 13.2 (4.7–11.7), and 703.8 (195.1–1371.8) Bq/kg, respectively. In South Carolina, USA, Powell et al., (2007) reported that the average activity (range) of ^238^U, ^226^Ra, ^232^Th, and ^40^K were 21.4, 45.3, and 609 Bq/kg, respectively. In contrast, the variation in activity concentration for ^226^Ra ranged from 62 to 633 Bq/kg (mean: 215 Bq/kg). ^232^Th contents ranged between 64 and 569 Bq/kg (mean: 131 Bq/kg). The ^40^K activity concentration ranged between 566 and 1139 Bq/kg (mean: 822 Bq/kg) in the stream sediments of Wadi El Reddah [45]. The average concentrations were 25 Bq/kg for ^226^Ra, 77 Bq/kg for ^232^Th, and 710 Bq/kg for ^40^K in Nigeria [46].

The radium equivalent (Ra_eq_) of granitic rocks ranged from 51.8 to 771 Bq/kg (mean: 320 Bq/kg) and for red and black silica veins in the El-Missikat area, the Ra_eq_ was reported as 68.5–6983 Bq/kg (mean: 1824 Bq/kg) [41]. Ra_eq_ had a positive correlation with both ^226^Ra and ^238^U. This was in accordance with the direct positive relationship between the concentration of ^38^U and ^226^Ra with Ra_eq_ for the investigated rocks in the El Sela area [60].

The results for the absorbed dose rate in the air (D) for the investigated granitic rocks ranged in values from 25.4 to 352.4 nGy/h. On the other hand, monzogranite showed the highest values of 352.4 nGy/h, while the average of red and black jasperoid veins was 836.8, with values ranging from 33.7 to 3215.5 nGy/h, which recorded high values, especially in red and black jasperoid veins in the El-Missikat area [41].

The gamma activity index (*I_γ_*) values ranged from 0.20 to 2.60 (mean: 1.10) for granitic rock samples. It was observed that all the samples had a gamma index *I_γ_* < 2, which indicated the gamma dose contribution from these sediment samples did not exceed 0.3 mSv/y. In contrast, in red and black jasperoid veins, the values ranged from 0.2 to 23.3 with an average value of 6.1, which was higher than the acceptable level of gamma dose in the El-Missikat area [41].

Values of internal hazard index (H_in_) ranged from 0.20 to 3.40 (mean: 1.30) for the granitic rocks samples of the El-Missikat area, while in the jasperoid veins, the H_in_ values ranged from 0.20 to 37.0 (mean: 9.40), which were higher than the acceptable level. On the other hand, the H_ex_ values ranged from 0.10 to 2.10 (mean: 0.90) for the granitic rocks samples, while in the red and black jasperoid veins, the H_ex_ values ranged from 0.20 to 18.9 (mean: 4.90), which were higher than the acceptable level [41].

When compared to the concentrations of ^238^U (1–6 ppm) and ^232^Th (1–23 ppm) by [61], ^238^U (3.00) and ^232^Th (8.00–17.0) by Turekian in 1961 [62] and ^238^U (5 ppm) and ^232^Th (18–20 ppm) by Clark (1966), the present results showed that the studied microgrants were highly enriched with ^238^U and ^232^Th. The values of ^238^U and ^232^Th in the study area are more than the concentrations of ^238^U and ^232^Th in the Um Taghir area, (Awad H. A. et al., 2020) and less than the concentrations in the El-Missikat area (Awad H. A. et al., 2020).

The natural radioactivity concentrations and radiological impacts of these samples confirmed the high trend of activity. The southeast Baroud area had a high concentration of radioactive elements more than those of different regions in other areas. Additionally, radiological hazards were much higher than the worldwide average and those of the other literature for comparison. Additionally, the radium equivalent activity—Ra_eq_ = 8937 Bq/Kg was 24 times higher than that reported for the worldwide average limit. The absorbed dose rate (D) (3929 nGy/h) was also 68 times higher than the worldwide recommendations [49,63]. The average annual effective dose equivalent (AEDE_outdoor_; 4.82 mSv/y) was also 68 times higher than that (0.07 mSv/y) of the worldwide average. The average AEDE_indoor_ (19.3 mSv/y) was 48 times higher than that (0.40 mSv/y of the worldwide average [63] and those of the other literature, as shown in Table 3 [50,57]. The Commission of International on Radiological Protection [ICRP, 1994] have been shown the dose equivalent limit of annual effective 1 mSvy^−1^ for the individual members of the public and 20 mSvy^−1^ for the radiation workers. The present range (14,512–18,497) of the excess lifetime cancer risk (ELCR) was higher than the limit of 1.45 of the worldwide average [50,64]. All the radiological hazard results indicated the presence of very high rates resulting in high human health risks. This confirmed that this area is not radiologically safe, and care must be taken when working in microgranitic rocks from the southeastern part of Wadi Baroud.

## 6. Conclusions

The occurrence of radioactive mineralization, located to the south of Wadi Ras Baroud, was evaluated for the first time in Abu Hadeida microgranite. These rocks were considered as having high uranium and thorium. The uranium concentrations of the studied rocks ranged from 2647 to 4735 Bq/kg (mean: 3680 Bq/kg), while the thorium concentrations ranged from 3201 to 4018 Bq/kg (mean: 3635 Bq/kg). The Ra (eU) concentrations varied between 3082 and 3990 Bq/kg (mean: 3650 Bq/kg). The potassium contents in these rocks were high, reaching up to 6.3 wt%. The eTh/eU ratio showed that the mineralization was not only magmatic but was also related to hydrothermal alteration processes. The positive correlation between eU and eTh might be related to the accommodation of the two elements in the same minerals, such as thorite, uranothorite, Zircon, and xenotime. Uranium migrated during late evolution, with a 9.1% migration rate and redistribution during or after modification, based on the form (inward migration or outward migration) and quantity of mobilized uranium. Radiation risk indicators were calculated, and they were compared to the UNSCEAR committee and other global ranges. The results showed that the risk of radiations from the studied area was higher than the United Nations Scientific Committee on the Effects of Atomic Radiation (UNSCEAR) guideline limits. All the radiological hazard results indicated high human health risks. This confirmed that this area is not radiologically safe, and care must be taken when working on microgranitic rocks from the southeastern part of Wadi Baroud. According to the present findings, the study area had a high uranium content, making it ideal for uranium extraction for use in the nuclear fuel cycle.

## Figures and Tables

**Table 1:** The activity concentrations (Bq/kg) of ^238^U, ^226^Ra, ^232^Th, and ^40^K, and their ratios in the Abu Hadeida microgranite.

Activity	^238^U	^226^Ra	^232^Th	^40^K	U Chem. (Bq/kg)	Uc/Ur	eU/Ra	eU/eTh	eTh/eU
Sample No:									
M.S.1	3020	3082	3201	566	-	-	0.98	0.94	1.06
M.S.2	3430	3853	3646	1162	9943	2.90	0.89	0.94	1.06
M.S.3	3194	3604	3666	1203	7457	2.33	0.89	0.87	1.15
M.S.4	4288	3729	3836	1028	9943	2.32	1.15	1.12	0.89
M.S.5	4735	3990	3844	1082	14,915	3.15	1.19	1.23	0.81
M.S.6	3865	3977	3909	1619	10,689	2.77	0.97	0.99	1.01
M.S.7	2647	3145	3205	1255	12,429	4.69	0.84	0.83	1.21
M.S.8	3269	3517	3391	1038	24,858	7.60	0.93	0.96	1.04
M.S.9	4673	3952	4018	1379	9943	2.13	1.18	1.16	0.86
Mean	3680	3650	3635	1148	-	-	-	-	-

**Table 2:** Overall statistics of ^238^U, ^226^Ra, ^232^Th, and ^40^K activity concentrations, radium equivalent, absorbed dose rate, gamma index, activity utilization index, excess-lifetime cancer risk, annual gonadal equivalent, AEDE_indoor_, AEDE_outdoor_, H_ex_, H_in_, and Clark ratio. N = 9.

	Mean	SD	Skewness	Kurtosis	Minimum	Maximum
^238^U (Bq/kg)	3680	748	0.31	−1.35	2647	4735
^226^Ra (Bq/kg)	3650	346	−0.79	−0.76	3082	3990
^232^Th (Bq/kg)	3635	304	−0.47	−1.31	3201	4018
^40^K (Bq/kg)	1148	287	−0.56	2.03	566	1619
Radium equivalent (Ra_eq_ (Bq/kg)	8937	782	−0.66	−1.02	7704	9804
Absorbed dose rate (D_R_; nGy/h)	3929	345	−0.67	−1.00	3381	4309
Gamma index (*I_γ_*)	30.6	2.68	−0.66	−1.03	26.4	33.6
Activity utilization index (I)	148	13.2	−0.69	−0.94	127	162
Excess lifetime cancer risk (ELCR)	16,865	1482	−0.67	−1.00	14,512	18,497
Annual gonadal equivalent (AGDE)	26,834	2359	−0.67	−1.00	23,084	29,441
AEDE_indoor_ (µSv/y)	19,274	1694	−0.67	−1.00	16,585	21,140
AEDE_outdoor_ (µSv/y)	4819	424	−0.67	−1.00	4146	5285
H_ex_	24.1	2.11	−0.66	−1.02	20.8	26.5
H_in_	34.0	3.04	−0.72	−0.92	29.1	37.2
Clark ratio (^232^Th/^238^U)	1.00	0.03	−0.37	−1.65	0.95	1.04

**Table 3:** Comparisons of ^238^U, ^232^Th, ^40^K, radium equivalent (Ra_eq_), absorbed dose rate (D_R_), and AEDE of the present study with those reported in the literature.

Country	Region	^238^U (Bq/kg)	^232^Th (Bq/kg)	^40^K (Bq/kg)	Ra_eq_ (Bq/kg)	D_R_ (nGy/h)	AEDE_indoor_ (mSv/y)	AEDE_outdoor_ (mSv/y)	Type of Samples	Reference
USA	South Carolina	37.8	45.3	609	NA	NA	NA	NA	River sediments	Powell et al., (2007) [47]
Venezuela	Venezuelan coast	11.4	14.5	153	44.3	20.6	NA	NA	Marine sediments	Alfonso et al., (2014) [48]
India	Western Ghats	36.3	108	232	208	133	0.449	0.112	Soil samples	Manigandan and Chandar Shekar (2014) [49]
Egypt	El-Missikat area	162	72.6	702	320	147.5	0.723	0.181	Granitic rocks	Awad et al., (2020, 2021) [34,41]
Pakistan	Northern Pakistan	50.7	70.2	514	191	87.5	0.810	0.110	River’s sediments	Qureshi et al., (2014) [50]
Korea	Keum River area	65.7	91.1	1005	NA	NA	NA	NA	Stream sediments	Lee et al., (2009) [51]
Saudi Arabia	Qassim	9.30	12.3	535	68.1	35.2	0.173	0.040	Soil	El-Taher and Al-Zahrani (2014) [52]
Turkey	Northwestern Turkey	22.3	26.8	419	NA	84.3	0.104	NA	Soil samples	Kapdan and Karahan (2011) [53]
Iran	Tehran city	38.8	43.4	555	144	69.1	NA	0.080	Soil samples	Asgharizadeh et al., (2013) [54]
China	Eastern Sichuan	26.0	49	440	130	60.0	0.074	NA	Soil samples	Wang et al., (2012) [55]
Nigeria	Niger Delta	18.0	22	210	76	30.0	NA	0.037	Soil, sediment and water samples	Agbalagba and Onoja (2011) [56]
Palestine	West Bank	68.7	48	630	186	88.2	0.61	0.110	Soil samples	Dabayneh et al., (2008) [57]
Malaysia	Kuantan	6.57	10.6	41.0	24.9	11.2	NA	0.010	Soil samples	Kolo et al., (2015) [58]
Egypt	South Baroud	3680	3635	1148	8937	3929	19.3	4.819	Granitic rocks	Present study
Worldwide average		33.0	45.0	412	370	58.0	0.41	0.070	-	UNSCEAR (2008) [59]

Note: UNSCEAR = the United Nations Scientific Committee on the Effects of Atomic Radiation. NA = not available.

## Data Availability

The authors declare that all data and materials are available to be shared on a formal request.
